# Applications of CRISPR screening to lung cancer treatment

**DOI:** 10.3389/fcell.2023.1295555

**Published:** 2023-12-15

**Authors:** Wanying Shen, Fangli Hu, Pan Lei, Yijun Tang

**Affiliations:** ^1^ Department of Neurosurgery, Taihe Hospital, Hubei University of Medicine, Shiyan, Hubei, China; ^2^ Hubei Hongshan Laboratory, College of Biomedicine and Health, Huazhong Agricultural University, Wuhan, China; ^3^ Hubei Clinical Research Center for Precise Diagnosis and Treatment of Liver Cancer, Taihe Hospital, Hubei University of Medicine, Shiyan, Hubei, China

**Keywords:** CRISPR screening, lung cancer, treatment, drug resistance, Tuba-seq

## Abstract

Lung cancer is an extremely aggressive and highly prevalent disease worldwide, and it is one of the leading causes of cancer death. Deciphering intrinsic genetic mechanism, finding new targets, and overcoming drug resistance are the key to lung cancer treatment. High-throughput CRISPR screening has been extensively used to obtain the genes related to cancers including lung cancer. This review describes CRISPR/Cas9 or CRISPR/dCas9-based technologies for high-throughput screening. We summarize the applications of CRISPR screening technology in exploring the mechanism of lung cancer development *in vivo* or *in vitro*, overcoming drug resistance, improving the effect of immunotherapy, and discovering new therapeutic targets. This review highlights the potential of CRISPR screening in combination with tumor barcoding and high-throughput sequencing (Tuba-seq) to precisely quantify the impact of alterations in many tumor suppressor genes on lung cancer.

## 1 Introduction

Lung cancer, an extremely aggressive and highly prevalent disease worldwide, is the most frequently occurring cancer and the first leading cause of cancer death in men, and the second leading cause of cancer death in women next to breast cancer (Sung et al., 2021). Lung cancer can be divided into non-small cell lung cancer (NSCLC) and small cell lung cancer (SCLC), of which NSCLC accounts for the vast majority of all cases, and NSCLC can be divided into adenocarcinoma (40%), squamous cell carcinoma (25%), and others (Leiter et al., 2023). Traditional therapies such as surgery, chemotherapy, and radiotherapy are the first choice, but drug resistance and systemic toxicity cannot be avoided in chemotherapy. Emerging therapies, such as targeted therapy and immunotherapy, have relatively low toxicity, but their application is limited, and they are also prone to induce drug resistance (Zheng X. et al., 2023). Therefore, overcoming drug resistance or finding new targets is the key to the treatment of lung cancer.

Prokaryotic CRISPR (clustered regularly interspaced short palindromic repeats)/Cas adaptive immune system can facilitate RNA-guided site-specific DNA cleavage. Under the guidance of short RNAs, Cas9 nuclease can also induce precise cleavage of endogenous genomic loci in human and mouse cells (Cong et al., 2013). The CRISPR/Cas system is not only useful for single-gene perturbations, but can also be used for high-throughput screening such as pooled screens, which has been widely used to obtain the genes related to cancer (Zhou et al., 2023). Unlike other gene editing tools, CRISPR-Cas9 has the multiple advantages such as wider editing site, lower off-target effects, and lower cost (Zheng R. et al., 2023).

This review introduces CRISPR/Cas9 and CRISPR/dCas9-based technologies including CRISPR knockout (CRISPRko), CRISPR interference (CRISPRi), and CRISPR activation (CRISPRa) for high-throughput screening and summarizes their application in revealing the mechanisms of lung cancer development *in vivo* or *in vitro*, overcoming the drug resistance, improving the effect of immunotherapy, and discovering more new therapeutic targets.

## 2 CRISPR screening approaches

**FIGURE 1:**
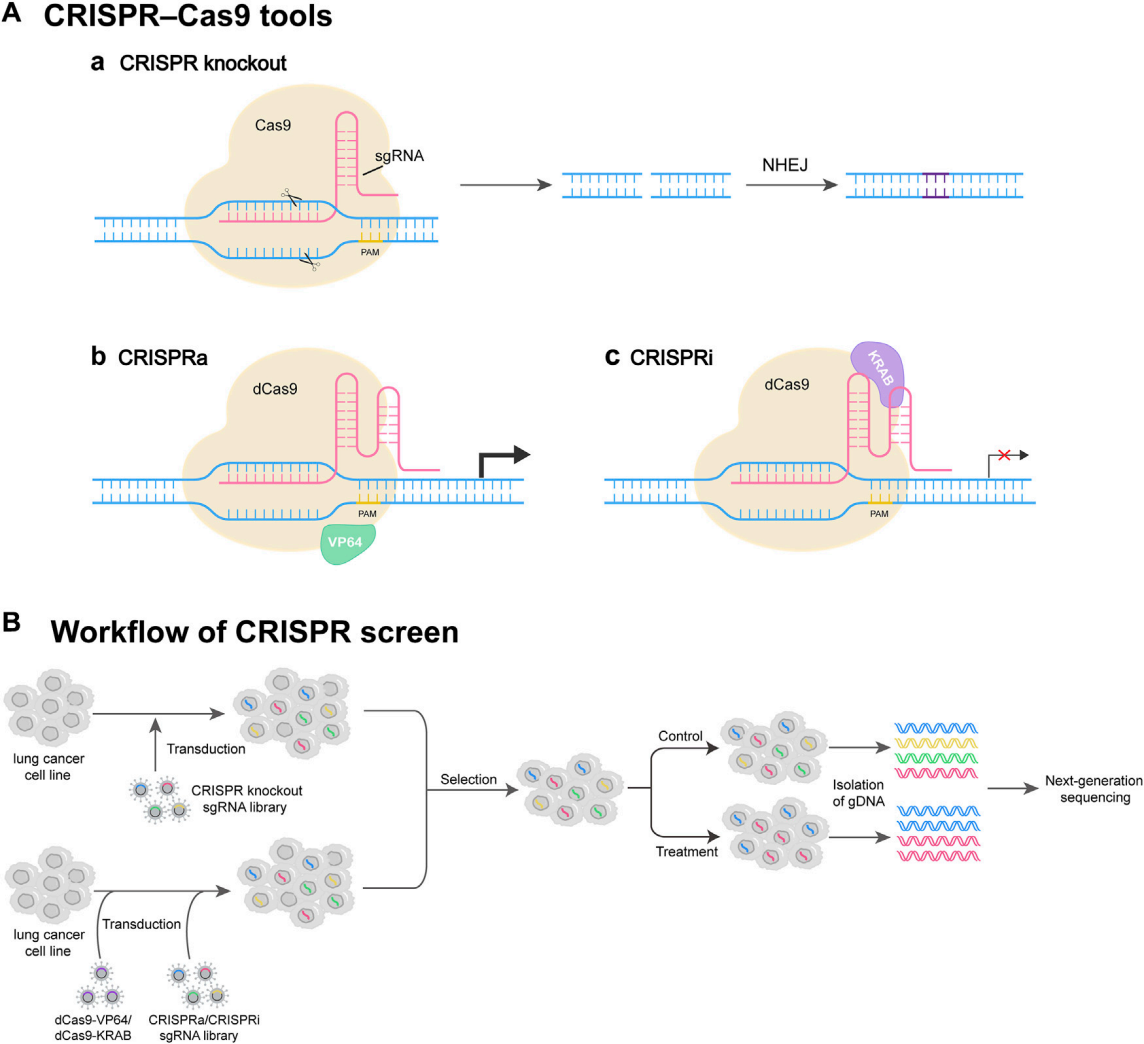
CRISPR screen tools and workflow. **(A)** Commonly used CRISPR perturbation methods. (Aa) CRISPR-Cas9 system consists of sgRNA and Cas9. sgRNA targets the gene of interest, and Cas9 induces a DNA double-strand break (DSB) at the target site. The DSB can be repaired by NHEJ, resulting in gene knockout. (Ab) CRISPRa system activates target gene expression through dCas9 fused with transcriptional activator VP64. (Ac) CRISPRi system represses target gene expression through dCas9 fused with transcription repressor protein KRAB. **(B)** Workflow of CRISPR screen. The lung cancer cells are transduced by a lentivirus-based CRISPR knockout sgRNA library, or by dCas9-VP64/dCas9-KRAB virus and CRISPRa/CRISPRi sgRNA library. Then, the cells successfully infected are selected and further propagated under control or treatment conditions. At appropriate times, gDNAs of cells are isolated, followed by next-generation DNA sequencing (NGS).

Traditionally, RNA interference (RNAi) technology is employed for genome-wide screening in mammalian cells. Compared to CRISPR screening, RNAi exhibits some limitations such as single function and off-target effects (Shalem et al., 2014). Currently, there are three CRISPR-based genome perturbation technologies (CRISPRko, CRISPRi, and CRISPRa) for screening (Figure 1). By designing sgRNAs and modifying Cas proteins, CRISPR technology enables flexible gene editing and screening (Shalem et al., 2014; Przybyla and Gilbert, 2022; Wang and Doudna, 2023).

### 2.1 CRISPR knockout screen

CRISPR RNA (crRNA) can hybridize with a small transactivating CRISPR RNA (tracrRNA), which is further recognized and bound by Cas9 to generate a ribonucleoprotein (RNP) complex. The RNP complex can bind to the genome to recognize the sequences matching the spacer region on the crRNA (Chavez et al., 2023). If there is a homologous sequence, Cas9 acts as a nuclease to cleave approximately 3 bp bases upstream the protospacer adjacent motif (PAM), causing a double-stranded DNA break (Hille et al., 2018; Liu et al., 2023). To simplify the above system, crRNA and tracrRNA are fused into a single guide RNA (sgRNA), which guides Cas9 to a specific gene locus to generate a double-stranded DNA break, highly possibly resulting in a error-prone repair via nonhomologous end joining (NHEJ) (Chavez et al., 2023; Zhou et al., 2023). NHEJ leads to insertions or deletions of bases at the cleavage site, thus achieving loss-of-function (LOF) mutations eventually resulting in disruption of the target gene (Shalem et al., 2015; Zhou et al., 2023).

Cas9 system has found various applications such as genome editing and large-scale screen (Chong et al., 2020). Ophir et al. (Shalem et al., 2014). First performed a genome-wide CRISPR/cas9 screen in human cells, confirming the feasibility of this technique. Genome-wide CRISPR screen in CD8 T cell-transferred mice in cancer immunotherapy setting identifies the regulators of tumor infiltration and degranulation and robustly reidentifies the regulators of canonical immune responses such as PD-1 and Tim-3. As a therapeutic target, a new gene *Dhx37* was discovered in T cells (Dong et al., 2019). Not only can the whole genome library be used for CRISPR screening, but also a specific sub-library can be constructed for screening according to the scientific questions to be studied. To screen RNA-binding proteins (RBPs) critical for macrophage activation, a lentiviral mini-library targeting 782 genes encoding known canonical RBPs in the mouse genome was constructed, and METTL9 was identified as a positive regulator of macrophage innate responses via CRISPR-Cas9 screen (Tong et al., 2021).

### 2.2 CRISPR interference screen

The dCas9, a nuclease-dead version of the protein, is generated from the removal of the catalytic activity of Cas9, and dCas9 can be used as a platform for transcriptional regulation (Chavez et al., 2023). The dCas9 can change transcription of any gene without genetically altering the target sequence (Qi et al., 2013). The dCas9 is bound with sequence-specific sgRNA to form the dCas9-sgRNA complex, and this complex can interfere with transcription elongation and hinder transcription initiation by disrupting transcription factor binding (Dominguez et al., 2016). The transcription repression function of dCas9 can be enhanced by linking the transcriptional repression domain Krüppel-associated box (KRAB) to dCas9 (Bendixen et al., 2023; Chavez et al., 2023).

CRISPR interference (CRISPRi) can inhibit the expression of pseudogenes without directly interfering with the transcription of parental genes. To investigate pseudogene function, the researchers designed the first genome-wide CRISPRi single-guide RNA (sgRNA) library targeting human pseudogene promoter-proximal regions for CRISPRi screening in breast cancer cells, and CRISPRi screening identified many pseudogenes that affect the fitness of breast cancer cells (Sun et al., 2021). Induced indels in open reading frames can disrupt translation of functional proteins, but the function of most noncoding genes is not affected, and large genomic deletions may affect nearby or overlapping genes (Goyal et al., 2017). In one previous study, CRISPR interference (CRISPRi) screen was used to identify MYC-regulated lncRNAs based on a library targeting sequences surrounding the transcription start sites (TSS) of non-coding genes, and CRISPRi screen successfully identified 320 non-coding sites that are vital for cell growth (Raffeiner et al., 2020).

### 2.3 CRISPR activation screen

In the past, genome-wide acquisition screening was performed using cDNA overexpression library, but the complexity of transcription isoform variation, long cDNA sequence, and high cost limit its application, and thus it is necessary to seek new techniques for genome-scale gain-of-function perturbation (Konermann et al., 2015). CRISPRa is one promising therapeutic gene editing strategy (Zhang et al., 2021). By fusing four copies of the transcriptional activator VP16 or one copy of the p65 activation domain (AD) to dCas9, the obtained dCas9-VP64 and dCas9-p65AD can effectively activate reporter gene expression (Gilbert et al., 2013). Compared with VP64, the second-generation activators (VPR, SAM, and Suntag) showed higher activation levels, mainly through the recruitment of multiple activation domains to the dCas9 complex (Chavez et al., 2016; Joung et al., 2017).

In addition to being used for the activation of individual genes, CRISPR activation can also be used for high-throughput screening. To evaluate lncRNA function, a CRISPR-activated lncRNA (CaLR) strategy was developed, and a library targeting lncRNA genes was designed to perform CRISPRa screening, resulting in the identification of new cell cycle regulators, survival/apoptosis and cancer signaling genes (Bester et al., 2018). A genome-scale CRISPRa screen was used to identify candidate genes related to the drug-resistance phenotype from PC9 cell lines under osimertinib treatment, and the identified genes could be new targets (Müller et al., 2023).

In addition to using a CRISPR screening method alone, multiple methods are combined for cancer treatment research. To systematically understand the regulators that coordinate T cell activation with gene perturbations, regulators responsible for cytokine production were discovered using genome-wide pooled CRISPRa and CRISPRi screens (Schmidt et al., 2022).

### 2.4 Others

In addition to traditional CRISPR-Cas9 mediated genome editing methods, two novel techniques, namely, base editing and prime editing, are gaining momentum in the field of genome editing. Base editing (BE) is a highly efficient and permanent technique for introducing single nucleotide variants (SNVs) into the DNA of living cells (Porto et al., 2020). BE does not require dependence on DSB, HDR, or a donor DNA template and it catalyzes the deamination of cytidine (C) or adenosine (A), which leads to the conversion of the bases to thymine (T) or guanine (G), respectively (Zhao et al., 2023). Base editor screening is a promising strategy for examining the functional effects of point mutations, which will contribute to genetic disease diagnosis and the identification of potential treatment strategies. Prime editing (PE) can create any kind of DNA substitutions, small insertions, and deletions at targeted sites within the genome of living cells, without relying on DSBs (Chen and Liu, 2023). PE consists of nCas9 (H840A) coupled to an engineered reverse transcriptase (RT) and prime editing guide RNA (pegRNA), which both matches the target site and encodes the required editing (Zhao et al., 2023). By sequencing integrated pegRNAs to identify genetic alterations, the prime editing system may also enable high-throughput pooled screens eventually (Chen and Liu, 2023).

## 3 Application of CRISPR screens in lung cancer treatment

**FIGURE 2:**
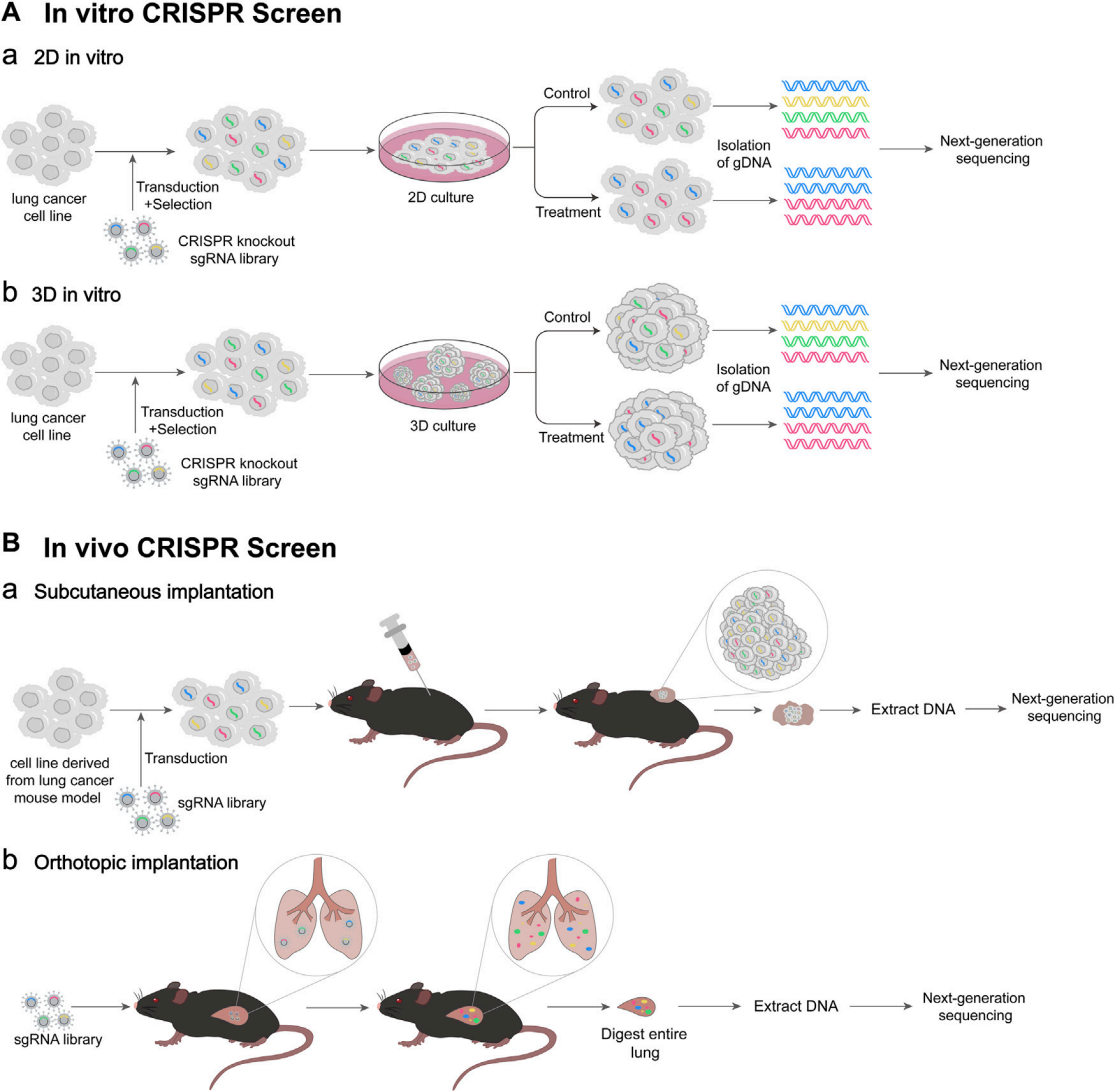
Schematic diagram of CRISPR screen. **(A)** Workflow of *in vitro* CRISPR screen. (Aa) 2D culture-based *in vitro* CRISPR screen. Lentivirus-based CRISPR knockout sgRNA library is transfected into lung cancer cells, and the cells successfully infected are selected. Then, 2D culture of cells is performed under control or treatment conditions. At appropriate times gDNAs of cells are isolated, followed by next-generation DNA sequencing (NGS). (Ab) 3D culture-based *in vitro* CRISPR screen. 3D culture of cells is performed under control or treatment condition. Then, gDNAs of 3D spheroids are isolated. **(B)** Workflow of *in vivo* CRISPR screen. (Ba) Cancer cell subcutaneous implantation. A lentivirus-based sgRNA library is transfected into lung cancer cells derived from lung cancer mouse model. The transfected cells are transplanted into mice subcutaneously. Tumor-derived DNA was extracted, followed by NGS. (Bb) Virus orthotopic inoculation. The lentivirus-based sgRNA library is delivered to mice lung intratracheally. After the entire mouse lung is digested, DNA is extracted, followed by NGS.

The CRISPR/Cas system is simple and efficient, and can modify multiple genes simultaneously, making it suitable for high-throughput genetic screening (Wang and Doudna, 2023). The above CRISPR screening methods can be used to explore the mechanism of the occurrence and development of lung cancer, overcome the drug resistance, improve the effect of immunotherapy, and discover more new therapeutic targets, and others (Figure 2) (Table 1).

**TABLE 1:** Reported CRISPR screens in lung cancer.

	Library	Stress	Model	Readout	Refs.
Overcoming chemotherapy resistance	Genome-wide CRISPR activation	EGFR inhibitor osimertinib	Human lung cancer PC9 cells	NA	Müller et al. (2023)
	Genome-wide knockout	cisplatin (DDP) or etoposide (VP16)	Human chemo-resistant SCLC cell line H69-AR	*In vitro* growth for 7 days	Deng et al. (2023)
	Brunello human genome-wide lentiviral gRNA pooled library	CHK1 inhibitor prexasertib	Human NSCLC cell lines A549-Cas9 and NCI-H460-Cas9	*In vitro* growth for 19 days	Branigan et al. (2021)
	Genome-wide CRISPR interference	CDK9 inhibitor and MCL1 inhibitor	Human lung cancer LK2 cells c-KRAB	NA	Kabir et al. (2019)
Overcoming radiation resistance	Genome-wide knockout	Radiation	Human NSCLC cell line A549	*In vitro* growth for 4 days	Cheng et al. (2021)
Boosting Immunotherapy	Mouse homologs of 573 human Genes associated with altered cytotoxicity in cancer	Treated with OT-I T cells at week 4 and immunized with OVA emulsified in CFA/IFA	Lox-Stop-Lox (LSL)-Kras^G12D^ or LSL-Braf^V600E^ mice	*In vivo* growth for 10 weeks	Dervovic et al. (2023)
	NA	anti–PD-1 antibody	KP cells were subcutaneously transplanted to C57BL/6 mice	*In vivo* growth for 24 days	Huang et al. (2022)
	524 epigenetic regulator genes	anti-PD-1	KP-Cas9-clone 7 library cells into Rag1^−/−^ and WT mice	*In vivo* growth for 24 days	Li et al. (2020)
Identifing novel targets	4,915 mouse genes corresponding to 5,347 human orthologs	NA	Cell lines derived from genetically engineered mouse models (GEMMs) of three cancers: SCLC (Trp53^−/−^; Rb1^−/−^), LUAD (Kras^G12D/+^; Trp53^−/−^), and PDAC (Kras^G12D/+^; Trp53^R172H/−^)	*In vivo* growth for 26 days	Li et al. (2019)
	Genome-scale knockout	NA	Lung cancer cell line, EKVX, for stable expression of Cas9	*In vivo* growth for 2 weeks	Liu et al. (2022)
	Genome-wide knockout	NA	Five cell lines derived from a genetically engineered autochthonous Rb1/Trp53-deleted SCLC mouse model	NA	Norton et al. (2021)
	Druggable-genome library targeting 4,915 genes	NA	KP tumor-derived cell lines	After 8 population doublings	Romero et al. (2020)

### 3.1 Overcoming chemotherapy resistance

Since genetic changes induced by drug treatment allow cells to acquire resistance or susceptibility, it is possible to screen relevant targets through CRISPRa-mediated gain-of-function and CRISPRi- and CRISPRko-mediated loss-of-function (Bodapati et al., 2020). Resistance to EGFR inhibitors (EGFRi) is a significant barrier to NSCLC treatment. CRISPR-based functional genomic screen is a new method to discover possible resistance indicators (Gogleva et al., 2022). In PC9 cells treated with osimertinib, a new potential driver of EGFR inhibitor resistance, WWTR1, was discovered by a genome-wide CRISPRa, and WWTR1 knockdown markedly decreased the development of drug-resistant cells (Müller et al., 2023). A kinome-wide CRISPR/Cas9 screen was performed in FGFR1-amplified lung cancer cells treated with FGFR inhibitors, identifying PLK1 as a potent synthetic lethal target for this cancer type (Yang et al., 2021).

A genome-scale CRISPR/Cas9 deletion screen in DDP- and VP16-treated and untreated resistant SCLC cells results in the identification of the serine/threonine kinase cell division cycle 7 (CDC7) as a promising target for chemotherapeutic synergy. The combined use of CDC7 inhibitors and other drugs helps to overcome chemoresistance in the treatment of SCLC and improves patient prognosis (Deng et al., 2023). A human-genome-wide library was employed to screen the genes necessary for CHK1i sensitivity in lung cancer cells, and several MMB-FOXM1 complex components were discovered (Branigan et al., 2021). Many models are naturally resistant to MCL1 inhibitor. A whole-genome CRISPR screen based on flow cytometry identified three components of the cullin-RING ubiquitin ligase complex CRL5 (CUL5, RNF7 and UBE2F), all of which resensitized cells to MCL1 inhibition (Kabir et al., 2019).

### 3.2 Overcoming radiation resistance

Radiotherapy is the cornerstone of cancer treatment, but radioresistance has always been a major obstacle, and the response rate of advanced NSCLC to radiotherapy remains unsatisfactory (Kim et al., 2020). Poly-ADP-ribose polymerase (PARP) inhibitors have been shown to radiosensitize SCLC tumors, and radiotherapy in combination with PARP inhibitor may be a promising therapy to overcome radioresistance (Laird et al., 2018; Cheng et al., 2021). CRISPR has been used to screen genes affecting radioresistance in glioblastoma (GBM) (Zhu et al., 2021), nasopharyngeal carcinoma (NPC) (Shen et al., 2021), and pancreatic cancer (PC) (Lan et al., 2022), providing new solutions for cancer treatment.

CRISPR screens have also been successfully applied in lung cancer treatment to discover genes responsible for radioresistance. A genome-scale CRISPR screen determined plakophilin 2 (PKP2) as a key driver of radioresistance in lung cancer cell line A549 cells by comparing sgRNA abundance (Cheng et al., 2021).

### 3.3 Boosting immunotherapy

A CRISPR/Cas9 screen was used in a mouse lung cancer model to assess the immunomodulatory potential of genes related to cytotoxicity alteration in human cancers. The results showed that the cancer testis antigen Adam2 was an immunomodulator and also an oncogene, and it could inhibit interferon and TNF cytokine signaling and reduce the presentation of tumor-associated antigens (Dervovic et al., 2023). Based on a mouse KP lung cancer model, an *in vitro* and *in vivo* CRISPR screening platform was constructed to thoroughly assess intracellular regulators of antitumor immunity. *Tsc2* was identified, and its deletion improved the response of tumors to ICB treatment (Huang et al., 2022). A genome-wide CRISPR screen based on fluorescence-activated cell sorting (FACS) identified the regulators of PD-L1 gene in the human NSCLC cell line NCI-H358, and PD-L1 gene was found to be efficiently induced when the heme biosynthetic pathway was disrupted (Suresh et al., 2020). A study investigated the roles of epigenetic genes in anti-tumor immunity using an epigenetic-focused CRISPR screen in the LUAD model, and discovered anti-silencing function protein 1 homolog A (*Asf1a*) as a possible therapeutic target (Li et al., 2020).

### 3.4 Identifing novel targets

CRISPR screen can be used to explore novel targets. In order to determine the impacts of loss of a single target on cell proliferation of LUAD, the researchers performed a CRISPR screening in eight LUAD cell lines. They found that *CASP8AP2* was a crucial activity factor regulating tumor cell death (Myacheva et al., 2023). Two genome-scale CRISPR/Cas9 screenings were conducted in the wild-type TP53 and RTK-containing cell lines with BEAS-2B cells serving as a control. The results showed that MDM2 was a potential therapeutic target (Wang et al., 2022). A genome-scale screen was used to identify tumor suppressor genes through knockout by CRISPR/Cas9 system. ZNF24 was found to be a powerful tumor suppressor in mouse xenograft and autologous lung cancer models as well as lung cell lines (Liu et al., 2022).

A library targeting potential druggable genes was used to conduct genetic CRISPR screening in tumor cell lines derived from mouse models. The results showed that the *de novo* pyrimidine biosynthesis pathway was critical target of SCLC. In several *in vivo* SCLC models, pharmacological inhibition of DHODH, one of the enzymes in this system, reduced tumor growth (Li et al., 2019). One previous study identified protein neddylation through a genome-wide CRISPR/Cas9 screen in five SCLC-derived cell lines from genetic animal models and mouse embryonic fibroblasts (MEFs) (Norton et al., 2021).

### 3.5 Others

CRISPR screen has also been used to explore the mechanism of lung cancer development. A genome-wide CRISPR/Cas9 screen identified *Cul5* as a crucial gene regulating SCLC metastasis in a SCLC spontaneous metastatic mouse model. Deletion of *Cul5* dramatically increased SCLC development by preventing integrin β1 degradation (Zhao et al., 2019). Additionally, CRISPR screens identified IGF1R and ERBB3 as key mediators of KEAP1-mutant cell growth (Vartanian et al., 2019). CRISPR-Cas9 is also used to screen potential tumor suppressors in an early-stage SCLC cell line. One of the top hits is MAX, which is mutated in human SCLC. MAX has been found to be an essential heterodimerization partner for MYC family proteins. *Max* deletion significantly speeds up SCLC progression by enhancing cell proliferation and transformation. In contrast, *Max* deletion stops tumor development in SCLC that overexpresses MYCL (Augert et al., 2020). A CRISPR screen reveals that histone demethylase UTX deficiency significantly promotes lung cancer progression (Wu et al., 2018).

Deletion of synthetic lethality target genes aggravates ROS-induced cell death in NRF2-hyperactivated NSCLC cells. CRISPR/Cas9 screens identified mitochondrial superoxide dismutase 2 (SOD2) as antioxidant enzymes genes in *KEAP1*-mutated NSCLC cell lines A549, and loss of SOD2 enhanced the efficacy of β-Lapafenone (Jiang et al., 2022). Domain-focused CRISPR screen is a method to identify key transcriptional regulators in cancer. A large-scale CRISPR interference (CRISPRi) screen in combination with epithelial-mesenchymal transition (EMT) and fate mapping by genetic tracing was employed to identify the genes involved in RTK signaling and epigenome regulation, resulting in the discovery of a large number of chromatin regulators and a limited number of kinome genes as regulators of EMT interconversion (Serresi et al., 2021).

## 4 Tumor barcoding and high-throughput sequencing

**FIGURE 3:**
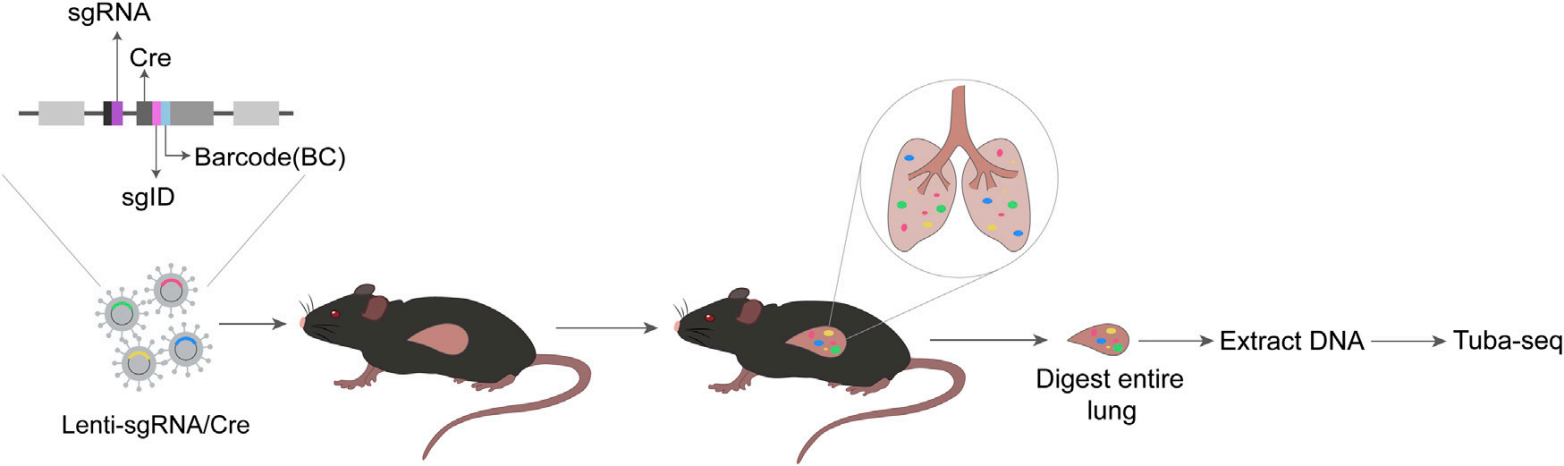
Schematic diagram of Tuba-seq. Each vector in the Lenti-sgRNA/Cre vector library contains a sgRNA, a Cre, and a two-component barcode sgID-BC consisting of an 8-nt ‘sgID’ corresponding to each sgRNA and a random 15-nt barcode (BC). The Lenti-sgRNA/Cre is delivered to mice intratracheally. At appropriate times, entire lung of mice is digested, and DNA is extracted. Then, Tuba-seq is performed, followed by data analysis.

Although many tumor suppressors have been identified by cancer genome sequencing, the strategies to test their *in vivo* functions in a quantitative manner are limited (Rogers et al., 2017). The tumor barcode and high-throughput sequencing enables high-resolution quantification of tumor growth in mouse models. Tumor barcode sequencing (Tuba-seq) and CRISPR technology when combined can utilize their unique strengths. The former provides precise measurement of tumor size whereas the latter modifies the expression of specific genes (Rogers et al., 2018). Two barcodes placed on the lentiviral vector, (one tracking TSG-targeting guide RNA (sgRNA), and the other tracking individual tumor), are used to measure tumor cell number and tumor size (Kim and Minna, 2018). The schematic diagram of Tuba-seq technology is shown in the figure (Figure 3).

On the one hand, the combination of Tuba-seq and CRISPR technology can more accurately characterize the effects of genetic alterations on tumor growth. Using CRISPR/Tuba-seq, a study of 48 TSGs characterizes the functional landscape of tumor suppression in an KRAS-driven lung cancer model, revealing novel genetics of KRAS-driven lung cancer initiation, overall and abnormal growth (Cai et al., 2021). A modified CRISPR/Tuba-seq method was used to quantify TSG function in EGFR-driven lung tumors and to identify key TSGs that limit the growth of *EGFR*-mutant tumors (Foggetti et al., 2021). Furthermore, multiple candidate KRAS-interacting proteins are studied for their effects on lung cancer growth in a genetically engineered mouse model using multiplex somatic CRISPR/Cas9 screenig combined with Tuba-seq. The results indicate that NRAS and HRAS act as tumor suppressor genes (Tang et al., 2023). Tuba-seq is suitable for the investigation of lentiviral transduction and condition-dependent regulation of oncogenic programs in *vivo* models, but it is not applicable to study the mechanisms underlying the natural development of SCLC. Considering this, a Lenti-Cre-based barcoded SCLC mouse model was developed to track tumors and investigate candidate regulators controlling tumor initiation and growth in their natural environment (Lee et al., 2023).

On the other hand, CRISPR/Tuba-seq allows for the analysis of how various genetic modifications affect therapeutic response, finding the therapeutic vulnerabilities, ultimately aiding in clinical drug guidance. The use of CRISPR/Tuba-seq indicated that KEAP1 inactivation considerably diminished the sensitivity of EGFR-driven lung adenocarcinoma to the EGFR inhibitor osimertinib in a TSGs pool (Foggetti et al., 2021). This study showed KEAP1 inactivation as a prominent resistance mechanism of osimertinib in lung adenocarcinoma. Another research employing CRISPR/Tuba-seq mapped a landscape of genotype-specific treatment responses and established over 20% of potential relationships between genotypes and treatment responses showing resistance or sensitivity (Li et al., 2021). Furthermore, CRISPR/Tuba-seq could also be used to find therapeutic vulnerabilities in lung cancer patients with no targetable proto-oncogenic mutations. Recent research through CRISPR/Tuba-seq have demonstrated that the inactivation of RAS and PI3K suppressors drives the development of lung tumor without oncogenic mutations and uncovered their therapeutic vulnerabilities (Yousefi et al., 2022). CRISPR/Tuba-seq contributes to the development of precision therapy by propelling the analysis of tumor genotypes and potential target identifications.

## 5 Conclusion

Loss-of-function CRISPR screens (CRISPRko and CRISPRi) and gain-of-function CRISPR screens (CRISPRa) are powerful tools that have facilitated comprehensive investigations of the genomic landscape of lung cancer. These approaches can be used to decipher the mechanism underlying the occurrence and development of lung cancer, overcome the cancer treatment resistance, improve the effect of immunotherapy, and discover more new potential therapeutic targets.

However, there are certain limitations with these approaches, such as off-target effects (Goyal et al., 2017), continuous technology innovation are expected to reduce the likelihood of off-target issues. Additionally, each CRISPR screen method has its own disadvantages. For instance, CRISPRko screen may not effectively identify genes critical for cell viability or those with functional redundancy (Chong et al., 2020). Hence, multiplexed screens are suggested to enhance the reliability and validity of research work.

In summary, the CRISPR/Cas9 system provides a potent approach for high-throughput screening. However, the limitations of this system should also be considered in order to obtain reliable results. To avoid false positives of CRISPR-based experiments, orthogonal techniques can be used for validation. In the future, CRISPR screening will play a prominent role in lung cancer research.
